# Clinicopathological Features of Telbivudine-Associated Myopathy

**DOI:** 10.1371/journal.pone.0162760

**Published:** 2016-09-09

**Authors:** Tomica Ambang, Joo-San Tan, Sheila Ong, Kum-Thong Wong, Khean-Jin Goh

**Affiliations:** 1 Division of Neurology, Department of Medicine, Faculty of Medicine, University of Malaya, Kuala Lumpur, Malaysia; 2 Department of Pathology, Faculty of Medicine, University of Malaya, Kuala Lumpur, Malaysia; 3 Department of Medicine, Queen Elizabeth Hospital, Kota Kinabalu, Sabah, Malaysia; Universidad de Navarra, SPAIN

## Abstract

Telbivudine, a thymidine nucleoside analog, is a common therapeutic option for chronic hepatitis B infection. While raised serum creatine kinase is common, myopathy associated with telbivudine is rare. Reports on its myopathological features are few and immunohistochemical analyses of inflammatory cell infiltrates have not been previously described. We describe the clinical, myopathological and immunohistochemical features of four patients who developed myopathy after telbivudine therapy for chronic hepatitis B infection. All four patients presented with progressive proximal muscle weakness, elevation of serum creatine kinase and myopathic changes on electromyography. Muscle biopsies showed myofiber degeneration/necrosis, regeneration, and fibers with cytoplasmic bodies and cytochrome c oxidase deficiency. There was minimal inflammation associated with strong sarcolemmal overexpression of class I major histocompatibility complex (MHC class I). Upon withdrawal of telbivudine, muscle weakness improved in all patients and eventually completely resolved in three. In our series, telbivudine-associated myopathy is characterized by necrotizing myopathy which improved on drug withdrawal. Although the occasional loss of cytochrome *c* oxidase is consistent with mitochondrial toxicity, the overexpression of MHC class I in all patients could suggest an underlying immune-mediated mechanism which may warrant further investigation.

## Introduction

Chronic hepatitis B virus (HBV) infection affects more than 360 million people globally and is a leading cause of liver cirrhosis, hepatocellular carcinoma and liver failure [1]. Of the two primary classes of antiviral therapy for chronic HBV infection, oral nucleoside/nucleotide analogs (NAs) have been found to be more effective than interferon-alpha/pegylated interferon-alpha [2]. To suppress HBV replication, NAs act by actively competing with endogenous substrates in viral DNA elongation and once incorporated act as chain terminators of viral DNA synthesis [3, 4]. Long-term NAs use has some drawbacks including the development of drug-resistant viral mutations and extra-hepatic serious adverse effects such as myopathy, nephropathy, neuropathy and lactic acidosis [4–7]. Clevudine was discontinued due to its myotoxicity while other NAs, including telbivudine and lamivudine, have been associated with raised serum creatine kinase (CK) and on rare occasions, clinical myopathy and even fatal rhabdomyolysis [3, 5, 7–11]. Furthermore, telbivudine has also been found to be associated with peripheral neuropathy especially when combined with pegylated interferon [5].

Nevertheless, NAs, including telbivudine, remain an important therapy and is the first line therapeutic recommendation in the current Asian-Pacific clinical practice guidelines on hepatitis B management [12]. The actual prevalence of telbivudine-associated myopathy is unknown, and reports of clinical myositis/myopathy from therapeutic trials for telbivudine did not have confirmatory muscle histopathology [13, 14]. In fact, there have only been a few muscle histopathology reports on telbivudine-associated myopathy, and none have provided any immunohistochemical (IHC) analyses of inflammatory cells involved [7, 9–11, 13–15]. We report the clinical and myopathological findings of four patients who developed myopathy after long-term administration of telbivudine for chronic HBV infection.

## Patients and Methods

### Patient characteristics

Four patients receiving telbivudine therapy for chronic HBV infection were referred to the Neuromuscular Clinic, University of Malaya Medical Centre (UMMC), Kuala Lumpur when they developed proximal muscle weakness and raised serum CK. As part of the standard investigative workup of patients with suspected acquired myopathy, they underwent clinical assessment, baseline blood tests, screening for myositis autoantibodies, electromyography (EMG) and muscle biopsy. All patients gave written informed consent for muscle biopsy and the study was approved by the medical ethics committee of UMMC (MECID. No: 20146–293).

### Muscle biopsy analysis

Biopsies were obtained from clinically-weak biceps brachii muscles from all four patients. The biopsies were snap frozen in isopentane cooled by liquid nitrogen, sectioned at 8 μM and stained with haematoxylin and eosin (H&E), modified Gomori trichrome, and a standard set of histochemical stains that included myofibrillar ATPase, nicotinamide adenine dinucleotide tetrazolium reductase (NADH-TR), acid phosphatase, cytochrome-*c* oxidase (COX), succinate dehydrogenase (SDH) and combined COX/SDH as previously described [16]. Standard IHC stains were used to identify CD4, CD8, CD20, CD68 cells and class I major histocompatibility complex (MHC class I) antigens (Dako, Glostrup, Denmark) as previously reported [17].

### Autoantibody profiling

Serum samples were analyzed for myositis-specific antibodies (MSA) and myositis-associated antibodies (MAA) using the commercial assay Myositis Profile 3 EUROLINE according to the manufacturer’s instruction (Euroimmun; Lübeck, Germany). Assays were interpreted using the EUROLineScan software as negative, borderline or positive (Euroimmun; Lübeck, Germany).

### Statistical analysis

Statistical analyses were performed using SPSS (IBM SPSS Statistics for Windows, Version 21.0. Armonk, NY: IBM Corp.).

## Results

The clinical features of our patients: two men, and two women, are summarized in Table 1. All four patients (age range: 60 to 73 years; mean 67.3 years) developed progressive proximal muscle weakness after 12 to 24 months (mean 17.3 months) on telbivudine therapy for chronic HBV infection. Muscle weakness was present for 2 to 17 months (mean 9 months) before they were diagnosed to have telbivudine-associated myopathy. Using the National Cancer Institute (NCI) of the National Institute of Health (NIH) Common Terminology Criteria for Adverse Events (CTCAE) version 4.03, the severity of generalized muscle weakness was grade 2 in three patients and grade 3 in one (Patient #2) [18]. This meant that they had symptomatic weakness, evident of examination, and limited their activities of daily living (ADL), and was disabling in Patient #2. Serum CK was elevated with the mean of 984.5 IU/L (range: 426 to 1798 IU/L). Two patients on simvastatin and one on atorvastatin had their statin treatment stopped when they developed muscle weakness at 3, 8 and 6 months, respectively, before telbivudine was discontinued. However, this did not result in improvement in muscle strength or reduction in serum CK levels. None had a family history of neuromuscular disease. All patients were shown to have myopathic EMGs but normal nerve conduction studies. The MSA and MAA were negative in three of the four patients in whom it was done.

**Table 1:** The clinical data of patients with telbivudine-associated myopathy.

	Patient 1	Patient 2	Patient 3	Patient 4
**Age at presentation (years)**	68	68	60	73
**Sex**	Male	Female	Female	Male
**Ethnicity**	Chinese	Malay	Chinese	Chinese
**Duration with HBV (months)**	23	26	240	240
**HBV DNA, IU/mL**	1120000	809200	66930	340000
**ALT (IU/L, normal 30–65)**	107	46	61	39
**AST (IU/L, normal 15–37)**	108	95	46	56
**Daily telbivudine dose (mg)**	600	600	600	600
**Duration of telbivudine therapy before symptom onset (months)**	20	12	13	24
**Interval between symptom onset and diagnosis (months)**	3	14	17	2
**Proximal limb weakness**	Present	Present	Present	Present
**EMG findings**	Myopathic	Myopathic	Myopathic	Myopathic
**Peak serum CK (IU/L, normal 39–308 (men) and 26–192 (women))**	1798	968	426	746
**MSA/MAA**	ND	Absent	Absent	Absent
**mRS score (at presentation)**	3	4	3	3
CTCAE* **(adverse events), grade**	2	3	2	2
**Statin therapy**	None	Simvastatin	Atorvastatin	Simvastatin
**Medication after diagnosis of telbivudine-associated myopathy**	Entecavir	Entecavir	Tenofovir	Entecavir
**Recovery time, months**	6	10	8	4
**mRS score (outcome)**	0	4	0	0
**Neurological outcome**	Normal	Poor	Normal	Normal

Abbreviations: ALT, alanine aminotransferase; AST, aspartate aminotransferase; CK, creatine kinase; CTCAE, common terminology criteria for adverse events; EMG, electromyography; HBV, hepatitis B virus; MAA, myositis-associated antibody; MSA, myositis-specific antibody; mRS, modified Rankin Scale; ND, not done.

*CTCAE grading for generalized muscle weakness are as follows:—Grade 1: symptomatic, weakness perceived by patients but not evident on physical examination; Grade 2: symptomatic, weakness evident on physical examination and weakness limiting instrumental ADL; Grade 3: weakness limiting self-care ADL, disabling.

**Fig 1:**
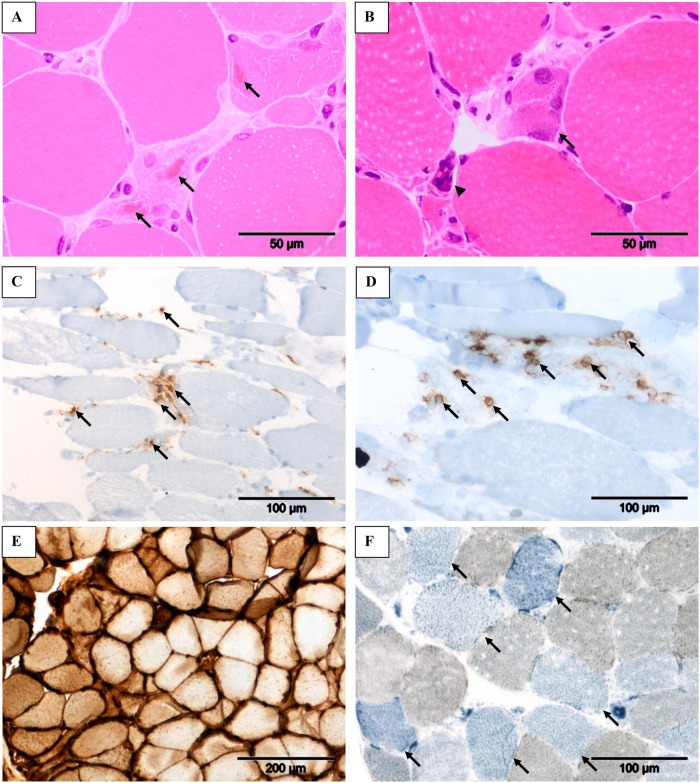
The myopathological features of our telbivudine-associated myopathy series. Cytoplasmic bodies (A, arrows) within degenerate/necrotic fibers, fiber regeneration (B, arrow) and clumped nuclei (B, arrowhead), and inflammatory infiltrates consisting mainly of CD4+ T-cells (C, arrows) and CD8+ T-cells (D, arrows) as observed in Patient 4. Strong sarcolemmal overexpression of MHC class I in Patient 1 (E) and COX-negative fibers (F, arrows) in Patient 4. Stains: Hematoxylin and Eosin (A & C), immunohistochemistry with 3, 3’ diaminobenzidinetetrahydrochloride chromogen/hematoxylin (C–E) and combined COX/SDH histochemistry (F). Original magnification: x 40 objective (A & B); x 20 objective (C, D & F); x 10 objective (E).

Muscle biopsy findings are shown in Table 2. The most prominent and consistent findings were fiber size variation, myophagocytosis, myofiber degeneration/necrosis and cytoplasmic bodies (Fig 1A), and fiber regeneration (Fig 1B). The inflammatory infiltrates consisted mainly of CD4+ (Fig 1C) and CD8+ T cells (Fig 1D). Additionally, all biopsies showed strong sarcolemmal overexpression of MHC class I antigens (Fig 1E) and occasional loss of COX activity (Fig 1F).

**Table 2:** The myopathological features of patients with telbivudine-associated myopathy.

Patients	Patient 1	Patient 2	Patient 3	Patient 4
**Hypertrophic fibers**	−	−	−	−
**Small rounded atrophic/angular fibers**	+	+	−	−
**Degeneration/necrosis**	+	+	+	+
**Regeneration**	+	+	+	+
**Clumped nuclei**	+	+	+	+
**Cytoplasmic body**	+	+	+	+
**Acid phosphatase activity**	+	+, Focal	−	+
**Myofibrillar disarray**	+	+	+	+
**COX-negative fibers**	+	+	+	+
**Inflammatory cell infiltration**†				
**CD4**	++	++	+	++
**CD8**	+	+	+	+
**CD20**	−	−	−	−
**CD68**	+	++	+	+
**MHC Class I**	Overexpressed	Overexpressed	Overexpressed	Overexpressed
**Endomysial fibrosis**	+	+	+	+

Abbreviations: +, present; −, absence; CD, cluster of differentiation; COX, cytochrome *c* oxidase; MHC class I, class I major histocompatibility complex.

^†^The presence of these cells are graded as “−” means negative, “+” means grade I positive (very minimal to minimal), “++” means grade II positive (mild to moderate).

Following the diagnosis of myopathy, telbivudine was discontinued and replaced with entecavir in three patients and with tenofovir in one (Table 1). Three patients recovered fully after a period between 4 to 7 months, while the other patient (Patient 2) showed only partial improvement of muscle strength and remained unable to stand and walk independently even at 10 months’ follow-up. She did, however, showed a reduction in CK levels to normal. In this patient, the diagnosis of myopathy had been delayed for 14 months and she would likely have had severe myofiber damage.

## Discussion

The actual prevalence of telbivudine-associated myopathy is unknown but in large telbivudine trials, this has been reported to be low [13, 14]. In the phase III GLOBE trial, despite 12.9% of patients on telbivudine developing severe elevation of serum CK levels (defined as more than seven times the upper limits of normal), clinical myopathy which was defined by the authors as having muscle symptoms in addition to raised serum CK, was reported only in two (0.3%) patients [13]. In another series of 200 chronic hepatitis B patients from China treated with telbivudine, the 3-year cumulative incidence of elevated serum CK was high at 84.3%, occurring more commonly in men than women, patients aged less than 45 years and those who were HBeAg-negative [14]. However, only nine patients (5%) were reported to have clinical myopathy and no risk factors for myopathy were identified [14]. Subsequent follow-up studies have shown that on-treatment, elevated serum CK levels were often transient and were not predictive of the development of myopathy [19, 20]. In our patients, CK elevations were mild to moderate only, further confirming that CK levels did not correlate with the development of myopathy.

There have been only a few reports of telbivudine-associated myopathy with detailed muscle histopathology findings [7, 15]. In a patient treated with telbivudine for a duration of 6 months after being given other NAs, including lamivudine and adefovir, muscle biopsy showed the presence of necrotic/degenerating and regenerating fibers, and on electron microscopy (EM), no mitochondrial abnormalities were seen [15]. On the other hand, in a series of six chronic HBV patients from China treated with NAs (three with telbivudine, three with lamivudine), muscle pathology was reported as showing non-specific abnormalities, including variation in fiber sizes, presence of angulated and regenerating fibers, and type I and II fiber atrophy, with necrotizing myopathy seen in only one patient on telbivudine [7]. In addition, all patients had positive oil red O staining suggesting an accumulation of lipids in their muscle fibers [7]. In contrast, all our patients had necrotizing myopathy with prominent muscle fiber necrosis and myophagocytosis. These findings were also previously seen in clevudine-associated myopathy [21].

NA-associated myopathy is considered to be due to mitochondrial toxicity [7, 21, 22]. In pre-clinical studies, telbivudine was not shown to have any effect on human DNA polymerase γ, a key enzyme involved in the mitochondrial DNA replication, and *in vitro* toxicity studies showed no mitochondrial abnormalities in human hepatocytes, skeletal muscle and neuronal cells [23]. However, in the case series from China, there was evidence of mitochondrial dysfunction, including the presence of ragged red (or blue) and COX-deficient fibers, mitochondrial abnormalities on EM and depletion of mitochondrial DNA in muscle [7]. Findings of COX-deficient fibers in our patients would support the mitochondrial toxicity of telbivudine, but as they are of the older age group, these findings could also be attributed to aging [24].

Interestingly, we also found overexpression of MHC class I antigens on the muscle fibers associated with inflammatory infiltrates consisting of both CD4+ and CD8+ T cells. Overexpression of MHC class I and CD8+ T cells has also been reported in human immunodeficiency virus patients with zidovudine-induced myopathy [22]. These features could alternatively suggest an underlying immune-mediated mechanism for NA-associated myopathy, besides drug induced mitochondrial toxicity. However, myositis autoantibodies were absent in our patients, and three of four patients recovered fully after drug withdrawal without any immunotherapy. The patient (Patient 2) who did not have complete clinical recovery nevertheless had her serum CK reduced to normal levels after discontinuation of telbivudine. Her incomplete recovery of muscle strength could indicate a more severe myonecrosis and poorer muscle fiber regenerative capacity.

In conclusion, telbivudine-associated myopathy presents as a sub-acute or chronic necrotizing myopathy with some evidence of mitochondrial toxicity as its underlying mechanism. As the overexpression of MHC class I antigens and the presence of inflammatory cells were the prominent and consistent findings in our patients, the possibility of a secondary immune-mediated inflammation may need further investigation.
